# Differential cytokine profiling in Chagasic patients according to their arrhythmogenic-status

**DOI:** 10.1186/s12879-017-2324-x

**Published:** 2017-03-21

**Authors:** Héctor Rodríguez-Angulo, Juan Marques, Ivan Mendoza, Marco Villegas, Alfredo Mijares, Núria Gironès, Manuel Fresno

**Affiliations:** 1 Instituto Venezolano de Investigaciones Científicas, Caracas, Venezuela; 2 Instituto de Medicina Tropical, Caracas, Venezuela; 3 Universidad de Castilla la Mancha, Ciudad Real, Spain; 4 Centro de Biología Molecular Severo Ochoa, CSIC-UAM, Cantoblanco, 28049 Madrid, Spain

## Abstract

**Background:**

Chagas disease is caused by the protozoan *Trypanosoma cruzi* and is characterized by heart failure and sudden death. Identifying which factors are involved in evolution and treatment response is actually challenging.

Thus, the aim of this work was to determine the Th1/Th17 (IL-6, IL-2, TNF, IL-17 and IFN-γ) and Th2 (IL-4 and IL-10) serum profile in Venezuelan Chagasic patients stratified according amiodarone treatment, hypertension and arrhythmias.

**Methods:**

Sera from 38 chagasic patients were analyzed to determine the level of cytokines by Multiplexed Bead-Based Immunoassays. ANOVA test was applied to determine differences for each group. Additionally, a Linear Discriminant Analysis (LDA) was applied to observe the accuracy of different cytokines to discriminate between the groups.

**Results:**

The levels of several cytokines were significantly higher in the high-risk of sudden death and untreated group. LDA showed that IL-2, IFN-γ and IL-10 were the best cytokines for discriminating between high-risk of sudden death and untreated patients versus low-risk of sudden death, treated and control groups.

**Conclusions:**

High IL-2 levels seem to identify patients with high-risk of sudden death and seems adequate as treatment efficacy marker. To our knowledge, this is the first report about the anti-inflammatory role of the amiodarone in Chagas disease, suggesting an inmunomodulatory effect that may be exploited as coadjutant therapy in chronic Chagas disease.

**Electronic supplementary material:**

The online version of this article (doi:10.1186/s12879-017-2324-x) contains supplementary material, which is available to authorized users.

## Background

Chagas disease is a neglected disease caused by the intracellular protozoan *Trypanosoma cruzi*. Initially, it was confined to Latin American countries, but has now spread worldwide by immigration [1]. Besides, non-traditional vectors have been recently involved in transmission [2], raising the concern about the dissemination in non-endemic countries.

Chagas disease is characterized by an acute phase, generally asymptomatic or with mild unspecific symptoms, such as fever and hepatomegaly. Patients progress to chronic phase, being only a 30% symptomatic during this stage. Chronic disease is characterized by heart failure, arrhythmias and sudden death. Particularly, sudden death represents 60% of mortality during any stage. The victims often are younger than 58 years and asymptomatic before the final episode [3], which made challenging the finding of new prognosis markers in order to identify patients with high-risk of heart failure and sudden death. Besides, these hypothetical markers would allow the evaluation of the response to the different treatments applied to Chagasic patients.

Several risk stratification systems have been proposed for Chagas disease. Some authors have postulated a classification that combines radiographic and echocardiographic criteria with QRS morphology and appearance of ventricular tachycardia [4, 5]. Other stratification systems have focused their attention in the differential cytokine response among patients with and without cardiomyopathy. Some authors have reported that higher IL-10 expression was associated with better cardiac function, as determined by left ventricular ejection fraction and left ventricular diastolic diameter values [6]. Besides, on the same grounds, other works have postulated that reduced production of the cytokines IL-10 and IL-17 in association with high levels of IFN-γ and TNF correlates with the severity of the Chagas’ disease cardiomyopathy, and the immunological imbalance observed may be causally related with deficient suppressor activity of regulatory T cells that controls myocardial inflammation [7]. In experimental animal models, IL-17 controls the resistance to acute *T. cruzi* infection regulating the Th1 cells differentiation, cytokine and chemokine production and control parasite-induced myocarditis, regulating the influx of inflammatory cells to the heart tissue [8]. However, our previous results suggested that IL-17-producing T helper (Th)17 cells may protect susceptible mice at low levels of infection, but could, in association with IL-6, be pathogenic at high parasite loads triggered by *T. cruzi* infection, whereas regulation of the Th1 response by regulatory T cells play a protective role in non-susceptible mice [9]. In the same direction, this pro-inflammatory response needs to be balanced in order to avoid tissue damage [10].

Despite these antecedents, there is little information about the cytokine profile in patients under risk of sudden death. This information is important in order to identify a valuable prognosis marker and to evaluate the response to the antiarrhythmic and heart failure therapy. These considerations should be taken intoaccount because there are several reports about the role of certain cardiovascular drugs in the modulation of heart inflammation in Chagas disease. It has been reported that captopril, an anti-hypertensive drug, was able to ameliorate myocarditis in acute experimental Chagas disease [11], although others have reported that decreased the expression of the modulatory cytokine IL-10 and development of the pro-inflammatory Th17 subset in human monocytes [12]. On the other hand, amiodarone, an antiarrhythmic drug, has been reported with anti-parasitic activity [13] and is able to improve the clinical outcome in Chagasic patients [14]. Interestingly, in the large >BENEFIT clinical trial, recently released, the only patients showing some clinical benefit were those taking amiodarone along with bendnidazole [15]. Nonetheless, reports about the possible role of amiodarone in the regulation of inflammation in Chagas disease are scarce. Thus, the aim of this work was to determine the Th1/Th17/pro-inflammatory and Th2/anti-inflammatory (serum profile in Venezuelan Chagasic patients stratified according to blood pressure status and Lown classifications and treated or not with amiodarone to evaluate progression and the response to treatment.

## Methods

### Human sera

Chagasic patients were diagnosed with two different serological tests (ELISA IgG and indirect haemagglutination), according to WHO criteria. Patients aged 26 to 72 years (mean 56.87), coming from North-West Venezuelan rural and urban areas, and the presence of other infectious disease (AIDS, TBC, Leishmaniosis, Toxoplasmosis), age under 18 years and above 75 were an exclusion criteria. Patients were recruited in a national specialized reference centre for studying Chagasic arrhythmias in Caracas, Venezuela. Following this diagnostic test results, patients were grouped in healthy controls (*n* = 10, serology negative for *Trypanosoma cruzi* and the same criteria of exclusion applied for Chagasic patients), cardiac chagasic positive (*n* = 38). Patients were not paired by sex (27 female and 11 male), due to response of patients to the service dating centre was not under the control of researchers. Samples were taken by conventional venepuncture performed by trained personal, serum was obtained by centrifugation and stored at −80 until use. Cardiac Chagasic positive patients were characterized following Lown criteria (Lown 0–2 *n* = 17, 3–5 *n* = 9) to evaluate their ectopic ventricular activity. Data of patients with amiodarone treatment or with cytokine data values out of distribution of the rest of data were excluded (Additional file 1). According to this classification, “0” stage represent patients without ectopical activity and “5” to patients with R on T phenomena (malignant ventricular arrhythmias). It should be noted that is considered that patients in 0–2 classifications are in low-risk (*n* = 17) and 3–5 classification are in high-risk (*n* = 9) of sudden death (low- and high-risk SD henceforth). Data outliers were excluded in the same say stated above. Finally, positive patients were divided regarding to Amiodarone treatment. For this classification, only were considered patients treated (*n* = 7, day by day at dosage of 200 mg daily five times a week) and untreated with a similar Lown grade (*n* = 14). Clinical data was obtained retrospectively and Amiodarone was prescribed based in conventional clinical criteria (presence of symptoms and reduced ejection fraction) independently of this study and treatment classification was independent of sudden death risk.

### Cytokine quantitation

Fluorescent bead-based flow cytometry assays for 7 anti-inflammatory and pro-inflammatory cytokines (CBA Human Cytokine assays, BD Biosciences) were performed in duplicate with human sera diluted 1:4, following the manufacturer’s directions. Briefly, seven bead populations with distinct fluorescence intensities coated with capture antibodies specific for IL-2, IL-4, IL-6, IL-10, TNF, IFN-γ, and IL-17A proteins were mixed together to form the bead array, which was resolved in FL4 channel.Sample reading was performed in BIO FACS Canto IITM Becton Dickinson (BD) cytometer and analyzed with FCAP Array™ v1.0.1 for Windows. After acquiring 30,000 events/microwell. Data was converted to pg/ml using one phase association fit curves, as is showed in Additional file 1.

### Semi-quantitative analysis

The cytokine profile was first assessed by identifying low and high cytokine producers, with slight modification from as previously reported [16]. Briefly, threshold for patients from all different groups was determined as the value were 70% of sensitivity for each group in curves ROC. These values were used as the cut-off mark to label each patient as being a high or low cytokine producer. Then, in each group and for each cytokine, the results were expressed as the frequency of individuals with a concentration of serum cytokine higher than the threshold of group samples. The graphs were plotted according to control frequencies in ascendant order. Finally, graphs of each group in ascendant order were overlaid to evidence cytokine signatures defined as those cytokines with frequencies above the 50%. Additional information about data distribution was added on Additional file 1.

### Multivariate statistical analysis

A multivariate ANOVA was performed in order to compare individually the variables studied. A Linear Discriminant Analysis (LDA) was applied to observe in which proportion the levels of cytokines were actually able to discriminate the study groups. LDA relates a variable measured in nominal scale (dependent) with a group of continuous variables, reducing dimensionality while preserving as much of the class discriminatory information as possible. The model assigns to each variable an axis, from classical X, Y and Z to a transformed space. Space reduction is based on correlation tests, where model choose the less correlated variable (s), which contributes in group discrimination. Thus, this method allows determining the most contributing variables. In addition, it allows predicting the adscription of each individual to the different groups studied with a certain probability [17]. Differences among classes are estimated by mean of multivariate tests Wilks’ lambda, Pillai’s trace, Hotelling-Lawley trace and Roy’s largest root that compute, in general terms, the source of variance. Outliers were determined by ROUT method and excluded of the analysis. Briefly, the program fits a theoretical non-linear model with the data, were the outliers has little impact. Thus, it uses a new outlier detection method, based on the false discovery rate (FDR), to decide which points are far enough from the prediction of the model to be called outliers. FDR was fixed at value of 5%. Fisher distances was used for determine distances between calculated centroids.

## Results

The levels of serum cytokines were determined in different groups of patients including non-chagasic controls and cardiac chagasic patients with different clinical status per blood pressure, and Lown classifications and in those presenting symptomatic treatment of arrhythmias or not with amiodarone.

**Fig. 1:**
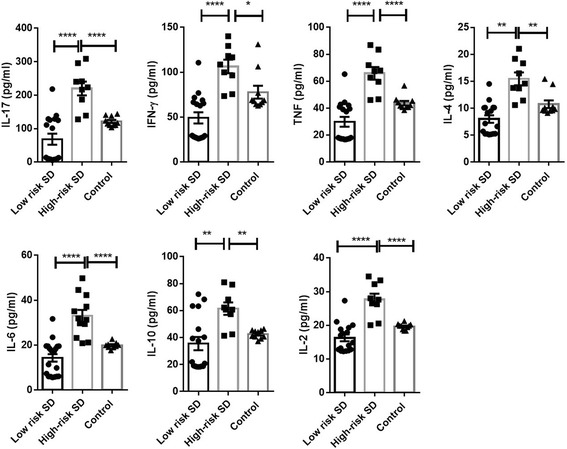
Cytokine levels for patients stratified following Lown criteria. Graphs show the mean +/− SEM values of values (expressed in pg/ml) for IL-17, IFN-γ, TNF, IL6, IL-2, IL-10 and IL-4 to Lown scoring classification (showed as risk of sudden death and non-arrhythmic uninfected control in bars). Significance both any group is marked with *, ** and *** in function of *p* values (*: >0.05 < 0.02; **: >0.02 < 0.01 and ***: >0.01). N value was 26 for Lown classification (17 low-risk vs. 9 high-risk). Male/female ratio were 4/5 for high-risk group and 5/12 for low-risk group and age mean were 56.222+/− 5.191 (high-risk group) and 53.588 +/− 11.138 (low-risk group). For additional information, the reader is referred to M&M section and Additional file 1

**Fig. 2:**
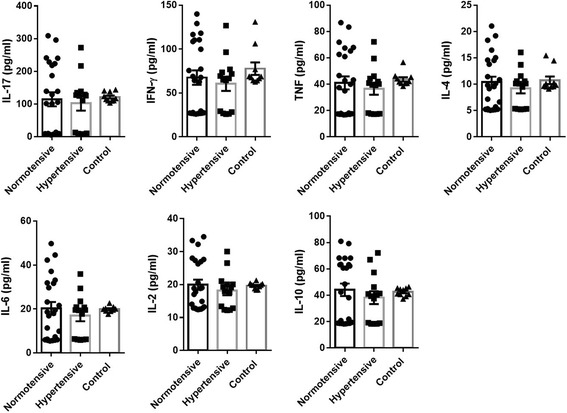
Cytokine levels for patients classified according Blood pressure status. Graphs show the mean +/− SEM values of values (expressed in pg/ml) for IL-17, IFN-γ, TNF, IL6, IL-2, IL-10 and IL-4 to Blood pressure classification (showed as normotensive, hypertensive and normotensive non-chagasic controls in bars). Significance both any group is marked with **, ** and *** in function of *p* values (*: >0.05 < 0.02; **: >0.02 < 0.01 and ***: >0.01). N value was 38 for blood pressure classification (14 hypertensive vs. 24 normotensive). Male/female ratio was 4/10 for hypertensive group and 7/17 for normotensive group and age mean were 57.286 +/− 6.170 (hypertensive group) and 55.583 +/− 10.579 (normotensive group). For additional information, the reader is referred to M&M section and Additional file 1

**Fig. 3:**
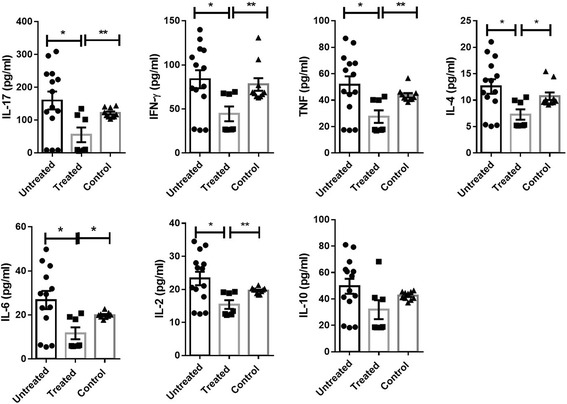
Cytokine levels for patients classified according amiodarone treatment. Graphs show the mean +/− SEM values of values (expressed in pg/ml) for IL-17, IFN-γ, TNF, IL6, IL-2, IL-10 and IL-4 to amiodarone treatment classification (showed as treated, untreated and untreated non-chagasic controls in bars). Significance both any group is marked with **, ** and *** in function of *p* values (*: >0.05 < 0.02; **: >0.02 < 0.01 and ***: >0.01). N value was 21 for item treatment classification (7 treated vs 14 untreated). Male/female ratio was 2/5 for treated group and 4/10 for untreated group and age mean were 61.143 +/− 6.440 (treated group) and 57.786 +/− 4.995 (untreated group). For additional information, the reader is referred to M&M section and Additional file 1

When stratifying the patients according to the Lown classification, which is based on ventricular arrhythmias, in high- and low-risk of sudden death (high- and low-risk SD), a significant increment in all the cytokines studied was observed in the high-risk SD group, but not in the low-risk SD, respect to control group (Fig. 1). Different cytokine relative values of control and chagasic patients stratified respect blood pressure in hypertensive and normotensive are shown in Fig. 2. The ANOVA statistical analysis indicated no significant differences between the groups. Most interestingly, patients treated with amiodarone for arrhythmias presented a significant decrease respect to the untreated ones in the relative levels of most of the cytokines analyzed (Fig. 3). Additionally, the percentage of high producer patients was estimated as the value of patients above the threshold determined by ROC analysis for each classification (Additional file 1). IFN-ƴ and IL-17 showed the highest percentages of high producers for Lown classification in patients with high risk of sudden death and, interestingly amiodarone treated group showed a global decrease of high producer levels.

Linear discriminant analysis (LDA) was applied to find a linear combination of variables, in this case cytokines that characterize two or more classes of situations or events, determining the variable (s) with the higher discriminatory ability. In addition, the analysis could be a predictive value for new individuals added to the different categories studied. In this case, the analysis was performed to find a function that allows the separation of the clinical groups where the individual differences for each cytokine were unable to show significant differences.

**Fig. 4:**
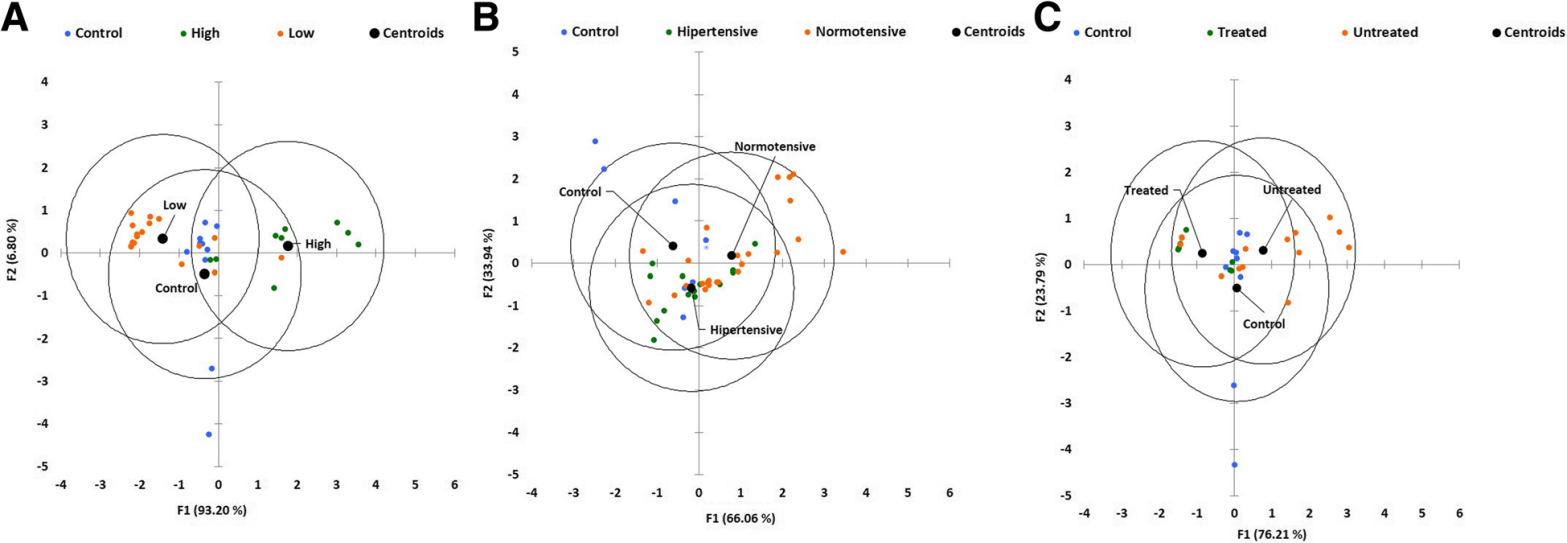
LDA analysis box plot for Lown (**a**), Blood pressure (**b**) and treatment classification (**c**). Axes represent the discriminant functions (F1 and F2) resulting from redundancies analysis and the variation conserved for each one onto the transformed space is showed in brackets for each discriminant function. Centroids (filled black circles, group pointed with continuous line) and confidence ellipses (unfilled black ellipses) are showed for each group. Finally, each small coloured circle represents individual data for each classification, as can be appreciated on the top of each axis

Striking differences in multivariate tests were observed according to the Lown classification (Fig. 4a). Despite some points generated in the transformed space are slightly dispersed, the centroids were significantly separated for all tests (Table 1). Fisher distances (Table 2) shows that the differences are centered between high-risk SD and low-risk SD groups, between high-risk SD and control groups and between control and low-risk SD. The function was able to predict retrospectively 77.78% members of high-risk SD, 70.59% for low-risk SD and 80% in the control group, with a global power of prediction of 76.12% (Table 3). Notably, LDA identified IL-2, IFN-ƴ and IL-10 as the most contributing variables for the discrimination of the Lown clinical stages (Table 4).

**Table 1:** LDA multivariate significance tests

	Wilks’ Lambda test	Pili’s trace	Hotelling-Lawley trace	Roy’s greatest root
Blood pressure	0.0587	0.0543	0.0673	0.0379*
Lown	< 0.0001*	0.0001*	< 0.0001*	< 0.0001*
Treatment	0.0054*	0.0052*	0.0075*	0.0040*

Multivariate tests from LDA that show comparison of vector means. Significance (*p* values >0.05) implies that at least one of the means vector is different from another. Significant values (*) are showed in bold

**Table 2:** Fisher distances among LDA calculated centroids

*p*-values for Fisher distances: Blood pressure			
	Control	Hypertensive	Normotensive
Control	1	0.2535	0.0464
Hypertensive	0.2535	1	0.1283
Normotensive	0.0464*	0.1283	1
*p*-values for Fisher distances: Lown			
	Control	High-risk SD	Low-risk SD
Control	1	0.0002*	0.0292*
High-risk SD	0.0002*	1	< 0.0001*
Low-risk SD	0.0292*	< 0.0001*	1
*p*-values for Fisher distances: Treatment			
	Control	Treated	Untreated
Control	1	0.0467*	0.0731
Treated	0.0467*	1	0.0050*
Untreated	0.0731	0.0050*	1

Fisher Distances Each subsection shows *p* values (significance >0.05, (*) bold when appear) from calculated Fisher distances among LDA centroids for sub-groups of the clinical classification used in this study. The *p* value corresponds to the intersection between column and rows variables

**Table 3:** LDA confusion matrix

Confusion matrix for the estimation sample: Blood pressure					
from \ to	Control	Hypertensive	Normotensive	Total	% correct
Control	5	8	3	16	30.00%
Hypertensive	2	9	5	16	57.14%
Normotensive	2	7	7	16	45.83%
Total	9	24	15	48	44.33%
Confusion matrix for the estimation sample: Lown					
from \ to	Control	High-risk SD	Low-risk SD	Total	% correct
Control	10	0	2	12	80.00%
High-risk SD	3	9	0	12	77.78%
Low-risk SD	3	1	8	12	70.59%
Total	15	10	11	36	76.12%
Confusion matrix for the estimation sample: Amiodarone treatment					
from \ to	Control	Treated	Untreated	Total	% correct
Control	4.13	0.00	6.20	10.33	40.00
Treated	4.43	5.90	0.00	10.33	57.00
Untreated	2.95	2.21	5.17	10.33	50.00
Total	11.51	8.12	11.37	31.00	49

LDA calculates membership probabilities an assign a group a posteriori in function of the higher probability obtained. Table shows the percentage of correspondence of the assignments compared with *a priori* known membership

**Table 4:** Summary of variables selection

Group	Variable	Lambda	F	DF1	DF2	*p*-value
SD-Risk	IFN	0.4841	17.5871	2	33	< 0.0001
SD-Risk	IL-2	0.3719	27.8637	2	33	< 0.0001
SD-Risk	IL-10	0.6091	10.5889	2	33	0.0003
Treatment	IFN	0.7142	5.6031	2	28	0.0090
Treatment	IL-2	0.6751	6.7385	2	28	0.0041

Mode stepwise for LDA discriminates the role of each variable studied in discriminate among the different group of Chagasic patients. Both for Lown (arrhythmias) or amiodarone treatment, LDA identified IL-2 as the best discriminator

Multivariate tests on blood pressure stratified patients showed significant differences for Roy’s greatest root and Fisher distances tests (Tables 1 and 2). Although the variation was lower in this classification (Fig. 4b), the centroids of the groups of patients were too close to each other (Table 1). Finally, the discriminant function was not able to separate *a posteriori* with and high accuracy Chagasic normotensive patients (45.33%) Chagasic hypertensive (57.14%) and control (30%) (Table 3).

The plot for amiodarone treatment classification shows the calculated centroids for each group (Fig. 4c). In this case, LDA was able to differentiate differences among treated group regarding to control and untreated ones (Table 1 and 2). The confusion matrix (Table 3) shows that the predictor function was able to separate *a posteriori* treated (57%), untreated group (50%), and control group (40%). Besides, Table 4 shows that LDA determined IL-2 and IFN-ƴ as the most discriminatory variable for treatment.

## Discussion

One of the biggest challenges in Chagas disease’s research is the searching for prognosis markers that help to determine which patients are in potential risk to develop the most devastating pathological consequences of the disease. On the other hand, comprehension of the pathophysiological processes involved in such evolution can lead to the design of a better rational therapy for improving the outcome of the patients during the chronic phase.

With the appearance of more sensitive techniques for detecting parasite antigens or DNA antigens in chronic lesions [18] it was thought that the parasite antigens persistence may play a role in the persistence of inflammation and the progression of pathology [19]. Besides, some studies associate Beznidazole treatment, a parasiticide drug, with better outcome in chronic Chagasic patients [20]. However, the recent report on the BENEFIT study that involved 2854 Chagasic patients from different geographic regions, failed to demonstrate any significant association between Beznidazole treatment and better clinical outcome [15], bringing back the concern about identifying factors involved in disease progression. Interestingly, in this study, the only group that appeared to benefit from therapy was the amiodarone treated group, but without any apparent effect on parasitic load [15, 21]. In this direction, we decided to explore the cytokine profile of Chagasic patients stratified by clinical status for sudden death risk (Lown classification), blood pressure status (hypertensive *versus* normotensive) and Amiodarone treatment, to discriminate the concomitant conditions that can influence the patient’s inflammatory status and, consequently, the outcome of the disease.

Interestingly, high-risk SD patients showed a significant overall increase of cytokine levels associated with increased high-producer frequencies. All multivariate tests and Fisher distances were able to find differences among centroids, especially between high- and low-risk SD groups. LDA predicts a very high percentage of high- (77.78%) and low-risk SD (70.59%), which strongly suggests association between inflammation and arrhythmia and postulate cytokine profiles as a plausible predictor of arrhythmias in Chagasic patients. Malignant arrhythmias, often asymptomatic, are the leading cause of death in Chagasic patients [22] and, in the best of our knowledge; this is the first report that associates cytokine profiles with arrhythmias in Chagas disease and it could open a field for better understanding the pathophysiology of this disease.

The role of inflammation in the genesis of arrhythmias is still and elusive issue in pathophysiology of sudden death. Infliximab, an TNF blocker, could reduce the frequency of mice afflicted by arrhythmias and second degree atrioventricular blocks in an experimental model of chronic Chagas disease [23]. Gene expression of pro-inflammatory factors associated to inflammatory response (IFN-γ, transcription factor T-bet, GATA-3; FoxP3 and CTLA-4; IL-17 and IL-18) were upregulated in heart samples of chronic Chagasic patients [24] and its inflammatory environment has been reported as inductor of gene expression related with heart failure [25] and gap junction dysfunction during Chagas disease [26], possibly explaining this fact the electrical disturbances observed in patients. Interestingly and closely related with our results, IL-2 was able to induce in vitro the expression of SCN3B and sodium current density [27], increasing of atrial action potential duration [28] and IL-2 has been linked to prognosis for atrial fibrillation in patients [29]. Additionally, Cx43 gene expression, a key protein of gap junctions tightly related with action potential spreading onto the heart, has been reported as impaired in Chagasic cardiomyopathy, reinforcing the possible association between inflammation and altered electrical function in Chagasic patients [30] and its fact possibly explains the positive effect of amiodarone treatment during Chagas disease [31]. Taken together, our results suggest that the cytokines, specially the pro-inflammatory ones, play a key role in arrhythmias and sudden death and may be explored in patients as a potential risk factor of malignant arrhythmias.

On the other hand, the serum cytokine profile showed in the present work cab be related with previous report of cardiac inflammatory milieu. TNF, IL-2, IL-10 and IFN-ƴ has been reported as locally produced by the inflammatory infiltrate in samples of human Chagasic hearts [32, 33], suggesting that may play a role on the differential susceptibility to chronic Chagas disease development. Other works have described the presence of IL-2, IL-4 and IL-6-producing mononuclear cells in Chagas heart tissue, associated with *T. cruzi* antigen presence [34]. In addition, recent publications have reported data showing that Chagasic patients with ventricular dysfunction had increased plasma levels of IL-10, IFN-ƴ, IL-6, TNF and IL1-β. Based on that, we may suggest that exist association among Th1/Th2/Th17 serum profile and *in situ* cardiac inflammation, reinforcing the profile proposed in this work as good marker of cardiac disease.

Normotensive Chagasic patients showed no significant differences in relative levels with respect to controls. Additionally, LDA analysis only found differences among centroids from control and normotensive group and has poor results for hypertensive Chagasic (57.14%) and control patients (30%) suggesting that hypertension was not associated with an inflammatory pattern in Chagasic patients. However, it is worth mentioning that anti-hypertensive drug treatment, although it was not a variable considered in this study, also decreased the levels of high producers in hypertensive patients (Additional file 1) [35]. Thus, it is probable that most of them were under anti-hypertensive treatment. Several drugs used for hypertension have been associated with anti-inflammatory properties [36] although in Chagasic patients the available data is contradictory [12]. Further studies are needed to resolve this issue.

On the other hand, LDA was able to identify IL-2 as the best discriminator variable, both in treated and untreated arrhythmic patients. Although this data does not allow itself to state that the increasing nor decreasing of IL-2 levels are associated to any outcome, high IL-2 serum levels have been reported as an atrial fibrillation predictor [29, 37] and low levels have been associated with therapeutic success of amiodarone treatment in atrial fibrillation [38]. Further studies are necessary for elucidating the molecular mechanisms associated to the heart microenvironment during chronic Chagas disease.

Finally, Amiodarone treated patients, paired with untreated Chagasic patients with the same arrhythmia classification, showed a decrease of cytokine levels and to levels like control group. It is interesting and could support the better prognosis observed in combination with beznidazol (in comparison with antiparasitic drug alone) reported by BENEFIT trial (Fig. 1 and Additional file 1). In a recent study, it was reported that amiodarone was able to inhibit in a dose dependent way the production of cytokines, IL-2, IL-4, TNF, and IFN-γ in activated human T cells through NFκβ and activate protein-1 modulation [39] and suppress the expression of IL-2 receptor-alpha (CD25) and CD69, cell surface markers of activated T cells [39]. Amiodarone has been reported to reduce polymorphonuclear leukocyte infiltration in the paw tissue and paw edema in a dose dependent way [40]. Neutrophil differentiation has been associated with increased IL-17 [41], suggesting that Amiodarone treatment may be associated with lower levels of IL-17 indicating lower Th17 lymphocyte response. Along those lines, it has recently been reported that Amiodarone treatment was able to improve heart failure prognosis in patients by decreasing IL-17 and IL-6 levels [42] which suggest that a pro-inflammatory state could be related with arrhythmias/heart failure and being benefited with amiodarone treatment. LDA was able to find significant distances among centroids from treated and untreated patients, but fails to predict the patients belonging to treated and untreated groups.

## Conclusion

Overall, a limitation of this study was the relatively low number for some groups of patients, especially those related with high scoring of arrhythmias. In the case of arrhythmias, it should be kept in mind the fact that it is a non-symptomatic alteration which generates a high sub-register for patients for high sudden death risk. Additionally, the diagnosis of arrhythmias often requires a very specialized medical evaluation and it is not always available for low incoming patients that are generally those mainly affected by Chagas disease. The pattern of patient affluence and the scarcity of centres specialized in arrhythmia diagnosis and treatment also limited the pairing of patients by sex and age. Although we did not observe significant differences between cytokine values discriminated by sex, nor correlation with age, it is necessary a larger scale study for elucidate the influence of these variables in cytokine levels. Despite those, the present work gives valuable insights on the relationship between inflammation and arrhythmias in Chagas disease. It also provides evidences about the role of Amiodarone as immunomodulatory agent in Chagasic patients, suggesting that it can help to improve the disease outcome, as recently demonstrated [15], through the mechanism described here. Last but not least, our results could help to propose a model of prognosis for evolution that is able to discriminate a characteristic profile for arrhythmic patients.

## Additional file

Additional file 1: Figure S1. One phase association fit curves for pattern curve data. Axes represent association between mean fluorescence (MFI) values obtained for different know cytokine with results expressed in pg/ml (y axis). Y=Y0 + (Plateau-Y0)*(1-exp.(-K*x)) equation was used for calculating cytokine concentration for patient samples. **Figure S2.** High producer frequencies for Lown stratified Chagasic patients. Bar graph shows the percentage of high producers for the different cytokines studied for Lown clinical scoring. Control high producer’s percentages is showed as dotted line and Chagasic frequencies (high & low SD risk) as bars. Threshold to determining high producers was calculated on ROC curve values for patients included in this group. **Figure S3.** High producer frequencies for blood pressure stratified Chagasic patients. Bar graph shows the percentage of high producers for the different cytokines studied for blood pressure clinical scoring. Control high producer’s percentages is showed as dotted line and Chagasic frequencies (normotensive & hypertensive) as bars. Threshold to determining high producers was calculated on ROC curve values for patients included in this group. **Figure S3.** High producer frequencies for amiodarone treatment stratified Chagasic patients. Bar graph shows the percentage of high producers for the different cytokines studied for blood pressure clinical scoring. Control high producer’s percentages is showed as dotted line and Chagasic frequencies (untreated & treated) as bars. Threshold to determining high producers was calculated on ROC curve values for patients included in this group. (XLSX 980 kb)

## Abbreviations

High-Risk SD: High risk for sudden death
IL: Interleukin
